# The Enhanced Recovery after Surgery Approach in Heart Valve Surgery: A Systematic Review of Clinical Studies

**DOI:** 10.3390/jcm13102903

**Published:** 2024-05-14

**Authors:** Pietro Giorgio Malvindi, Olimpia Bifulco, Paolo Berretta, Michele Galeazzi, Jacopo Alfonsi, Mariano Cefarelli, Carlo Zingaro, Hossein M. Zahedi, Christopher Munch, Marco Di Eusanio

**Affiliations:** 1 Cardiac Surgery Unit, Lancisi Cardiovascular Center, Ospedali Riuniti delle Marche, Polytechnic University of Marche, 60121 Ancona, Italy; 2 Cardiac Anaesthesia and Intensive Care Unit, Lancisi Cardiovascular Center, Ospedali Riuniti delle Marche, 60121 Ancona, Italy

**Keywords:** ERAS, cardiac surgery, heart valve, aortic valve, mitral valve, minimally invasive cardiac surgery

## Abstract

**Background:** Enhanced recovery after surgery (ERAS) protocols aim to reduce postoperative complications and promote earlier recovery. Although it is well established in noncardiac surgery fields, the ERAS approach has only recently been adopted in cardiac surgery. The aim of this review is to evaluate the status and implementation of ERAS protocols in patients undergoing heart valve surgery and to summarise associated clinical results. **Methods:** A literature search for the period January 2015 and January 2024 was performed through online databases. Clinical studies (randomised controlled trials and cohort studies) on patients undergoing heart valve surgical procedures and comparing ERAS and conventional approaches were included. The data extracted covered studies and populations characteristics, early outcomes and the features of each ERAS protocol. **Results:** There were 14 studies that fulfilled the final search criteria and were ultimately included in the review. Overall, 5142 patients were identified in the 14 studies, with 2501 in ERAS groups and 2641 patients who were representative of control groups. Seven experiences exclusively included patients who underwent heart valve surgery. Twelve out of fourteen protocols involved multiple interventions from the preoperative to postoperative phase, while two studies reported actions limited to intraoperative and postoperative care. We found high heterogeneity among the included protocols regarding key actions targeted for improvement and measured outcomes. All the studies showed that ERAS pathways can be safely adopted in cardiac surgery and in most of the experiences were associated with shorter mechanical ventilation time, reduced postoperative opioid use and reduced ICU and hospital stays. **Conclusions:** As demonstrated in noncardiac surgery, the adoption of structured ERAS protocols has the potential to improve results in patients undergoing heart valve surgery. Further evidence based on larger populations is needed, including more homogenous pathways and reporting further outcomes in terms of patient satisfaction, recovery and quality of life after surgery.

## 1. Introduction

Heart valve surgery is nowadays performed with a high safety profile, with real-world/national databases reporting a low rate of mortality after mitral valve surgery (~1%) [1] and for patients undergoing aortic valve replacement (<2%) [2]. Valve repair is largely performed in degenerative mitral valve disease, with evidence of excellent durability and freedom from symptoms, recurrence of mitral regurgitation and reoperation [3,4]. Aortic valve replacement also represents a safe treatment in elderly patients [5] and, in synergy with transcatheter procedures, can successfully address high-risk or technically demanding scenarios [6].

Having achieved excellent results in terms of safety and efficacy, new efforts have been put in place to reduce hospitalisation times and to promote prompt postoperative recovery after cardiac operations. Since the late 1990s, several experiences showed that fast-track programs, including the optimisation of intraoperative anaesthesia and targeting early extubation, were feasible and safe in cardiac surgery patients and allowed a reduction in ICU and hospital stays [7,8,9,10,11]. The establishment of a multidisciplinary approach that incorporates several actions and improvements throughout the entire surgical pathway—from the preoperative to postoperative phase—represents the core of enhanced recovery after surgery (ERAS). ERAS protocols aim to reduce postoperative complications and promote earlier recovery, as has already been demonstrated over the last decade in noncardiac surgery populations [12]. Although well established in general and thoracic surgery, this approach has only recently been adopted in cardiac surgery, with the first consensus guidelines only becoming available in 2019 [13].

The purpose of this narrative review is to evaluate the status and implementation of ERAS protocols in patients undergoing heart valve surgery; to summarise the aspects that have been targeted for improvement and optimisation in the preoperative, intraoperative and postoperative stages; and to report the surgical results in patients treated following an ERAS pathway compared to patients undergoing surgery following conventional perioperative care.

## 2. Methods

### 2.1. Definition

Enhanced recovery after surgery (ERAS) is a “multimodal, transdisciplinary care improvement initiative to promote recovery of patients undergoing surgery throughout their entire perioperative journey” aiming “to reduce complications and promote an earlier return to normal activities” [13]. ERAS programs incorporate multiple actions ranging from the preoperative to the postoperative period, including improving patients’ health status and physical and psychological conditions before surgery, providing a reduction in surgical tissue and biological trauma, enhancing pain control and ensuring a reduced mechanical ventilation time and early mobilisation aimed at rapid recovery and patients’ improved overall experience.

### 2.2. Literature Search

A literature search for the period January 2015 and January 2024 was performed through three online databases (PubMed, Medline, Google) according to the Preferred Reporting Items for Systematic Reviews and Meta-Analyses (PRISMA) criteria [14]. The following keywords were used: “*ERAS*” or “*enhanced recovery*” and “*cardiac surgery*”, “*valve surgery*”, “*aortic*”, “*mitral*”. The inclusion criteria were clinical studies (randomised controlled trials [RCTs] and cohort studies) on patients undergoing heart valve surgical procedures comparing conventional and ERAS approaches. Studies that included congenital or GUCH cardiac surgery, non-heart valve operation, non-cardiac surgery or transcatheter procedures were excluded. Similarly, comments, letters to the Editor, review articles, meta-analyses and conference abstracts were excluded. Studies with overlapping patient sets from the same institutions were reviewed to include only the largest or most recent data series.

Two reviewers (O.B. and M.G.) independently reviewed all records for inclusion and extracted data separately from relevant studies; divergences were resolved by consensus after discussion with a third reviewer (P.G.M.).

The data extracted from relevant studies included the following:

- Study period, number of patients, type of procedures;
- Patients’ characteristics: populations’ mean age, gender;
- Outcomes: early mortality (in-hospital or 30-day mortality), stroke, acute kidney injury, postoperative atrial fibrillation, postoperative pain management and use of opioids, time of mechanical ventilation, length of intensive care unit stay, overall postoperative hospital stay;
- Key actions included in the ERAS protocols.

In the case of studies that also included non-valvular procedures, we retrieved and presented data from patients who underwent heart valve surgery if provided separately. ERAS key actions were grouped according to the preoperative, intraoperative and postoperative phases. Data were presented as mean and standard deviation, as median and lower (Q1) and upper (Q3) quartiles or as proportions (percentage).

## 3. Results

### 3.1. Study Selection

We identified 2312 papers in the literature search, and their abstracts were reviewed. There were 14 studies that fulfilled the final search criteria and were ultimately included in the review [15,16,17,18,19,20,21,22,23,24,25,26,27,28] (Table 1). The PRISMA search flow diagram and checklist are available in Supplemental Figure S1 and Supplemental Table S1.

Overall, 5142 patients were identified in the 14 studies, with 2501 patients who underwent cardiac surgery following an ERAS protocol and 2641 patients representing control groups. One study was an RCT and included 209 patients [15], four studies provided propensity-match analyses [22,26,27,28] (2416 patients), and the remaining nine papers were observational cohort studies [16,17,18,19,20,21,23,24,25] (2495 patients). Seven experiences exclusively included patients who underwent heart valve surgery [20,21,23,24,26,27,28], while mixed case series were reported in the remaining papers with a prevalence of heart valve procedures ranging from 10% up to 50%.

### 3.2. ERAS Protocols

**Figure 1:**
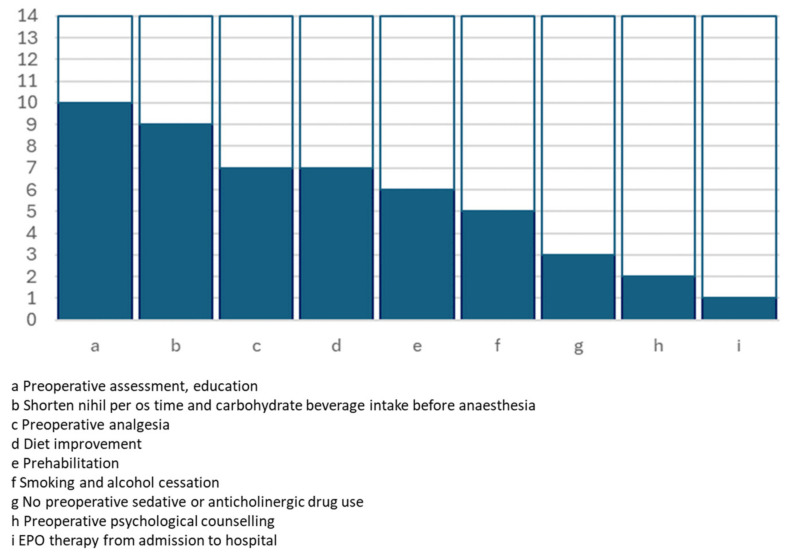
Graphical representation of the number of studies including preoperative key actions.

**Figure 2:**
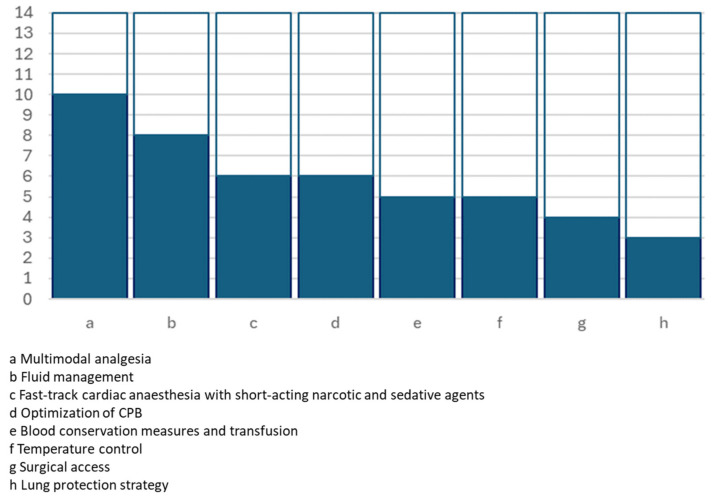
Graphical representation of the number of studies including intraoperative key actions.

**Figure 3:**
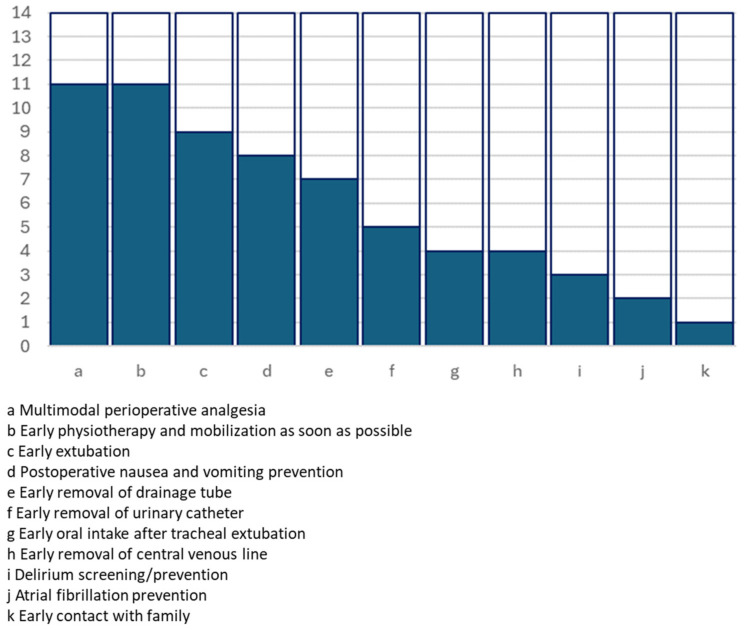
Graphical representation of the number of studies including postoperative key actions.

All the full texts provided details about the ERAS protocol that was adopted in each of the experiences. Table 2 and Figure 1, Figure 2 and Figure 3 summarise the key actions included in the proposed enhanced recovery pathways, which were furthermore grouped according to the preoperative, operative and postoperative phases.

**Table 1:** List of the full-text papers included in the review summarising patients’ characteristics, postoperative outcomes and key findings associated with the ERAS approach.

Author(s), Year	Study Period	Patients	Age (Years)	Sex (F/M)	Type of Valve Surgery	Mortality	Stroke	AKI	Postoperative AF	Time of Mechanical Ventilation (Hours)	ICU LOS (Hours)	Hospital LOS (Days)	Other(s)
Li et al., 2018 RCT [15]	2015–2016	104 ERAS	51.0 ± 10.1 (25.0–69.0)	53/51	Aortic n = 11 (10.58%), mitral n = 11 (10.58%)	0	0	2 (1.9%)	4 (3.8%)	7.2 (0.0–22.3)	20.9 (13.5–69.3)	6.0 (2.0–14.0)	ICU readmission 1 vs. 1 (ERAS vs. control) Reintubation 0 vs. 1 (ERAS vs. control)
		105 control	52.2 ± 10.4 (23.0–69.0)	56/49	Aortic n = 17 (16.19%), mitral n = 8 (7.62%)	0	1 (0.9%)	3 (2.9%)	12 (11.4%)	8.8 (3.7–44.9)	22.0 (13.4–212.3)	7.0 (4.0–16.0)	
			*p* = 0.36	*p* = 0.79		-	-	-	*p* = 0.04	*p* < 0.0001	*p* = 0.001	*p* = 0.07	
Blitzer et al., 2022 OS [16]	2019–2020	34 ERAS	51.7 ± 14.83	9/34	Valve surgery n = 15 (18%)	1 (3%)	0	-	5 (15%)	70.6 ± 287.0	165 ± 312	15.4 ± 14.7	
		49 control	54.3 ± 13.6	19/30		1 (2%)	0	-	6 (12%)	59.3 ± 136.6	165 ± 192	16.8 ± 14.4	
			*p* = 0.41	*p* = 0.24		*p* = 0.79	-	-	*p* = 0.75	*p* = 0.81	*p* = 0.99	*p* = 0.66	
Hendy et al., 2022 OS [17]	2019–2020 vs. 2017	100 ERAS	63.0 ± 10.7	18/82	Aortic n = 26 (26%), mitral n = 5 (5%)	0	0	0	13 (13%)	6.7 ± 1.6	-	5.1 ± 1.3 vs.	Time to ambulation (hours) 9.78 ± 2.03 vs. 40.43 ± 46.70 (ERAS vs. control) *p* < 0.001
		103 control	64.1 ± 9.7	34/69	Aortic n = 26 (25%), mitral n = 3 (2.9%)	0	1 (0.97%)	0	15 (14.6%)	14.7 ± 33.1	-	8.9 ± 3.5	
			*p* = 0.04	*p* = 0.02	-	-	*p* = 1	-	*p* = 1	*p* < 0.001	-	*p* < 0.001	
Fleming et al., 2016 OS [18]	2010–2011	52 ERAS	68.6 ± 11.1	14/38	Aortic n = 5 (9.6%), mitral n = 7 (13.5%)	1 (1.9%)	0	2 (3.8%)	7 (13.5%)	-	-	6 (4–7)	First postoperative intake of enteral solids day 1: 42 (80.1%) vs. 29 (54.7%) (ERAS vs. control) *p* = 0.007
		53 control	66.5 ± 11.8	15/38	Aortic n = 8 (15.1%), mitral n = 3 (5.7%)	2 (3.8%)	2 (3.8%)	6 (7.5%)	15 (28.3%)	-	-	6 (5–9)	
			*p* = 0.34	*p* = 0.87	*p* = 0.71	*p* = 0.57	*p* = 0.16	*p* = 0.27	*p* = 0.06	-	-	*p* = 0.31	
Williams et al., 2019 OS [19]	2017	443 ERAS	65 vs. 65 (ERAS vs. control)	31/34	Mitral/tricuspid = 15%, aortic = 17%	-	-	-	-	5.3 (3.9–6.9)	28 (23–52)	6 (5–8)	GI complications 3.6% vs. 6.8% (ERAS vs. control) (*p* = 0.04)
		489 control	65	31/34		-	-	-	-	5.2 (3.9- 7.3)	43 (25–74)	7 (5–9)	
			-	-		-	-	-	-	*p* = 0.53	*p* < 0.01	*p* < 0.01	
Zaouter et al., 2019 OS [20]	2014–2015	23 ERAS	80 (74–82)	14/9	Aortic valve (mini-sternotomy)	-	-	0	9 (39%)	-	24 (24–28)	7 (6.5–8)	Pulmonary infection 2 (9%) vs. 7 (30%) (ERAS vs. control) *p* = 0.06
		23 control	73 (68–82)	7/16		-	-	2 (9%)	6 (26%)	-	28 (25–47)	10 (9–13.5)	
			*p* = 0.16	*p* = 0.04		-	-	*p* = 0.15	*p* = 0.35	-	*p* = 0.003	*p* < 0.001	
Petersen et al., 2021 OS [21]	2018–2019	61 ERAS	50.7 ± 12.9	14/47	Aortic n = 37 (61%), mitral n = 24 (39%)	0	-	-	-	-	26.5 ± 25.2	6.1 ± 2.6	
		69 control	54.1 ± 9.5	17/52	Aortic n = 35 (51%), mitral n = 34 (49%)	0	-	-	-	-	46.6 ± 44.9	7.7 ± 3.8	
			*p* = 0.09	*p* = 0.81	*p* = 0.26	-	-	-	-	-	*p* = 0.01	0.008	
Yazdchi et al., 2021 OS [22]	2017–2019	76 ERAS	62.7 ± 9.7	25/51	Aortic n = 22 (29%), mitral n = 14 (19%)	0	0	0	11 (14.5%)	3.5 (3.1–4.7)	28 (23–47)	5 (4–7)	Reoperation for bleeding 0 vs. 0 (ERAS vs. control)
		76 control	63.2 ± 12.8	25/51	Aortic n = 21 (28%), mitral n = 12 (16%)	0	1 (1.3%)	1 (1.3%)	20 (26.3%)	5.3 (3.7–7.5)	48 (26–69)	6 (5–8)	
			0.76	*p* = 1	*p* = 0.4	-	*p* = 1	*p* = 1	*p* = 0.1	*p* = 0.01	*p* = 0.005	*p* = 0.03	
Bills et al., 2022 OS [23]	2019–2020	133 ERAS	65 (60–72)	41/92	All valve procedures	-	-	-	-	10.8 (9.3–12.9)	-	5.8 (4.9–7.1)	Opioid-related complications ERAS 57% vs. control 63%
		185 control	65 (58–71)	54/131		-	-	-	-	8.85 (7.3–10.3)	-	6.1 (5–8)	
			*p* = 0.59	*p* = 0.66		-	-	-	-	*p* = 0.47	-	*p* = 0.89	
Loria, 2022 OS [24]	2018 vs. 2020	216 ERAS	62 (51–70)	83/167	Valve n = 54 (22%)	8 (3.2%)	2 (0.8%)	8 (3.2%)	69 (27.6%)	4.9	74	6.5	Chest tube removal postoperative day 3 vs. 4 (ERAS vs. control) *p* < 0.0001)
		250 control	64 (57–70)	66/150	Valve n = 65 (30.1%)	5 (2.3%)	1 (0.5%)	6 (2.8%)	62 (28.7%)	4.7	3.3	6	
			*p* = 0.13	*p* = 0.54	*p* < 0.001	*p* = 0.77	*p* > 0.99	*p* = 0.79	*p* = 0.79	*p* = 0.54	*p* = 0.3	*p* = 0.5	
Gebauer et al., 2023 OS [25]	2018–2020	101 ERAS	56 ± 17	27/74	Aortic n = 51 (52%), Mitral n = 49 (49%)	-	2 (2%)	-	25 (24.8%)	-	18.5 ± 6	7 ± 3	-
		111 control	57.5 ± 13	32/79	Aortic n = 49 (44%), mitral n = 62 (56%)	-	2 (1.8%)	-	17 (15.3%)	-	26.5 ± 29	8 ± 4	
			*p* = 0.015	*p* = 0.73	*p* = 0.28	-		-	*p* = 0.08	-	*p* < 0.01	*p* < 0.01	
Giger et al., 2023 OS [26]	2015–2018 vs. 2018–2020	259 ERAS	69.7 ± 8.3	111/148	Aortic valve	1 (0.4%)	1 (0.2%)	5 (1.9%)	84 (32%)	6.9 ± 25	1.7 ± 1.8	7.7 ± 6.7	Mechanical ventilation time < 6 h 87% vs. 65% (ERAS vs. control) *p* < 0.001 Delirium 4 (1.5%) vs. 14 (5.4%) (ERAS vs. control) *p* = 0.028
		229 control	70.4 ± 9.7	114/115		1 (0.4%)	2 (0.4%)	1 (0.4%)	88 (34%)	6.5 ± 3.9	2.2 ± 3.1	7.4 ± 4.5	
			*p* = 0.39	0.79		*p* = 1	*p* = 0.56	*p* = 0.1	*p* = 0.79	*p* = 0.86	*p* = 0.039	*p* = 0.5	
Obafemi et al., 2023 OS [27]	2017–2018	747 ERAS	68.7 (61–75)	157/590	Aortic valve	25 (3.3%)	-	-	-	23.5 (9.6–122.6)	54.0 (40.4–97.0)	6.0 (4.9–8.8)	Days to first ambulation 1.6 (1.5–2.6) vs. 2.3 (1.6–3.5) (ERAS vs. Control) *p* < 0.001
		747 control	67.3 (59–75)	165/582		30 (4%)	-	-	-	272.4 (22.2–839.9)	69.9 (40.8–116.7)	7.0 (5.2–11.0)	
			*p* = 0.16	*p* = 0.61		*p* = 0.47	-	-	-	*p* < 0.001	*p* = 0.01	*p* < 0.001	
Berretta et al., 2023 OS [28]	2016–2020	152 ERAS	69.6 ± 11.1	84/78	Aortic n = 108 (71%) Mitral n = 44 (29%)	0	0	2 (1.3%)	41 (27%)	0	30 (24–52)	6 (5–7.7)	Respiratory insufficiency 1 (0.7%) vs. 5 (3.3%) (ERAS vs. Control) *p* = 0.04
		152 control	70 ± 11.9	78/84	Aortic n = 107 (70%) Mitral n = 45 (30%)	1 (0.7%)	2 (1.3%)	5 (3.3%)	49 (32.3%)	6 (4–9)	40 (24–59)	7 (6–8)	
			-	-		*p* = 0.9	*p* = 0.5	*p* = 0.4	*p* = 0.4	*p* < 0.001	*p* = 0.003	*p* = 0.04	

AF: atrial fibrillation, AKI: acute kidney injury, ICU: intensive care unit, OS: observational study, RCT: randomised controlled trial.

**Table 2:** Key actions included in the ERAS protocols grouped according to each perioperative stage and their level of evidence (LOE) as recognised by the latest Consensus Guidelines and Statement on ERAS in cardiac surgery.

Action	Details	N of Studies	References	LOE in Engelman et al. [13]	LOE in Grant et al. [29]
***Preoperative***					
Preoperative assessment, education	Personal meeting, video tutorial, pamphlet	10	[15,17,18,19,20,21,22,24,25,26]	IIa C	Moderate Low
Preoperative psychological counselling		2	[15,21]		
Prehabilitation	Pamphlet with exercises; in-person meeting with physiotherapist(s)	6	[17,19,20,21,25,26]	IIa B	Low
Diet improvement	High-energy, high-carbohydrate diet	7	[19,20,21,24,25,26,28]	IIa C	
Smoking and alcohol cessation		5	[17,19,22,26,28]	I C	
EPO therapy from admission to hospital		1	[15]		
Shorten nihil per os time and carbohydrate beverage intake before anaesthesia	Up to 2–6 h before anaesthesia	9	[15,16,17,18,19,22,25,26,28]	IIb C	Low
No preoperative sedative or anticholinergic drug use		3	[15,25,26]		
Preoperative analgesia	Paracetamol, gabapentin, pregabalin	7	[16,17,18,19,20,22,24]	I B	
***Intraoperative***					
Multimodal analgesia	Including locoregional analgesia: paravertebral block, infiltration at the incision site	10	[15,19,20,21,22,23,24,25,26,28]	I B	Moderate
Fast-track cardiac anaesthesia with short-acting narcotic and sedative agents		6	[15,17,19,22,25,28]	IIa B	Moderate
Optimisation of CPB	MIECC, fluid reduction, flow	6	[15,20,21,25,26,28]		Low
Lung protection strategy	Low tidal volume (6–8 mL/kg) ventilation, positive end-expiratory pressure, lung recruitment manoeuvre	3	[15,20,26]		High
Fluid management	Goal-directed (TOE-guided)	8	[15,19,20,21,22,24,25,26]	I B	Moderate
Blood conservation measures and transfusion	Cell saver, antiplasmin agent, antifibrinolytic and TEG monitor	5	[15,20,24,26,28]	I A	Moderate
Temperature control		5	[17,19,22,26,28]	I B	
Surgical access	Sternotomy and minimally invasive access	4	[20,21,25,28]		
***Postoperative***					
Early extubation	On table ICU early extubation	9	[17,19,20,21,22,25,26,27,28]	IIa B	Low Moderate
Multimodal postoperative analgesia	Patient-controlled analgesia, regional analgesia, infiltration at incision site	11	[15,17,18,19,21,22,23,25,26,27,28]	I B	Moderate
Postoperative nausea and vomiting prevention	Usually i.v. ondansetron	8	[15,16,17,18,19,22,25,26]		Moderate
Atrial fibrillation prevention		2	[16,21]		Moderate
Delirium screening/prevention		3	[20,24,28]	I B	High
Early oral intake after tracheal extubation	Usually from 6 h since extubation	4	[15,17,26,28]		
Early removal of drainage tube	No clear criteria	7	[15,20,21,22,24,25,26,28]		
Early removal of urinary catheter	No clear criteria	5	[20,21,22,25,26]		
Early removal of central venous line	Venous line removed at discharge from ICU/removed approximately after 12 h	4	[20,21,22,26]		
Early physiotherapy and mobilisation as soon as possible	From 3 h since extubation. Different protocols including chest physiotherapy, bed activities and full mobilisation within 12–24 h	11	[14,15,17,18,19,20,21,22,25,26,27,28]		Moderate
Early contact with family		1	[28]		

### 3.3. Preoperative Phase

#### 3.3.1. Assessment, Education and Psychological Counselling

Ten studies contemplated actions regarding patient’s assessment and counselling in their preoperative ERAS protocol [15,17,18,19,20,21,22,24,25,26]. The education of patients and families was usually carried out a few days or weeks before the hospital admission with an in-person meeting involving nurses or within a multidisciplinary team including all the healthcare figures who were expected to take care of the patient throughout the treatment journey. This included a detailed explanation of their pathologies, the perioperative care, the expectations from treatment and recovery. Booklet and video tutorials were used to facilitate communication and to reduce stress and anxiety by presenting the hospital environments and each step of the perioperative course [20]. Two studies provided evidence of the implementation of preoperative psychological counselling conducted by specialised and trained staff [15,21].

#### 3.3.2. Prehabilitation and Diet Improvement

Six of the fourteen studies focused on prehabilitation before heart valve surgery [17,19,20,21,25,26]. Usually, this period started 2–3 weeks before surgery and included explanation and education on how respiratory exercises are performed properly, and the recommendation for daily training until admission [17,20,21,25,26]. None of the papers reported details regarding the type of exercises and the availability of different protocols based on the patients’ functional status and underlying pathologies. Most of these experiences provided in-person meetings with specialised personnel (nurses, physiotherapists, surgeons, anaesthetists) [20,21,25,26].

Screening of nutritional status and counselling was reported in three protocols [19,20,25]. Four experiences recommended a high-protein [19,26] or high-energy, high-carbohydrate diet [21,25] as supplemental nutrition from 1 week to 2 weeks before hospital admission.

The promotion of smoking cessation and improvement of diet before surgery was contemplated by all the studies supporting a prehabilitation period [17,19,20,21,22,25,26,28].

#### 3.3.3. Preoperative Fasting

Several experiences recommended shortening the nihil per os period and allowed the consumption of clear fluid until 2–4 h before surgery [15,17,18,19,22,25,26,28] and solid food until 6–8 h preoperatively [19,26,28]. A carbohydrate drink of 200 mL or 400 mL was prescribed 2–6 h before surgery [15,16,17,18,19,22,25,26] as an adjunct to maintain gut motility and to be used as an energy source (protein-sparing effect) [17].

#### 3.3.4. Preoperative Analgesia

Multimodal analgesia has been embedded in most of the studies included in this review [15,19,20,21,22,23,24,25,26,28], with wide heterogeneity according to the choice of drugs and the timing and dose of administration. Seven protocols contemplated preoperative analgesia starting before the transfer to the operative theatre, aiming for a reduction in opioid use in the postoperative period [16,17,18,19,20,22,24]. This pre-emptive analgesia usually included paracetamol (acetaminophen), gabapentin or pregabalin.

### 3.4. Intraoperative Phase

#### 3.4.1. Chest Wall Analgesia

Locoregional analgesia was provided with different protocols and included paravertebral blocks [15,26,28], performed at T2–3 and T5–6 levels with the injection of 8–10 mL of 0.25% ropivacaine at each site before the induction of anaesthesia [15]; transverse thoracic plane and anterior serratus plane block [26,28]; local infiltration at the incision and drains insertion sites [15,20,24,28]; the continuous delivery of local anaesthetic through a fascial catheter during the first 24 h [25,28].

#### 3.4.2. Fast Track Anaesthesia

Six studies provided a clear statement regarding the adoption of fast-track cardiac anaesthesia with short-acting narcotic and sedative agents [15,17,19,22,25,28].

Hendy et al. [17] provided details about their protocol including the induction of anaesthesia after 3 min of preoxygenation followed by IV injection of sufentanil (0.5–1 mcg/kg), ketamine (0.5 mg/kg), propofol (1–2 mg/kg) and rocuronium (1.5 mg/kg) sequentially; the maintenance of anaesthesia in the pre-bypass period using a continuous infusion of sufentanil 0.2–0.6 mcg/kg/h and sevoflurane anaesthetic gas (1.5–2.5%) to achieve a minimum alveolar concentration of 0.8; during the bypass time, sevoflurane was turned off and propofol was infused at 80–150 mcg/kg/min instead. Sufentanil infusion (0.2–0.6 mcg/kg/h) was continued until the end of surgery.

Williams et al. [19] administered i.v. fentanyl, typically <1 mg, for the entire case and hydromorphone (0.5–1 mg) near the completion of surgery.

Yazdchi et al. [22] used a 0.2 mcg/kg/hour sufentanil infusion started after induction and discontinued on transfer from the operating room to the ICU. A 0.5 mcg/kg bolus of sufentanil was given prior to sternotomy. Gebauer et al. [25] induced the anaesthesia with sufentanil (50 μg) and propofol (1.5 mg kg^−1^) and neuromuscular blocking with rocuronium (0.6 mg kg^−1^). The maintenance of anaesthesia was achieved with remifentanil (0.4–0.5 μg kg^−1^ min^−1^), propofol (2 mg kg^−1^ h^−1^) and a variable sevoflurane concentration (end-tidal Vol.% 0.6–1.8). On CPB, sevoflurane was administered via the CPB circuit.

#### 3.4.3. Cardiopulmonary Bypass Management

Six studies included actions regarding cardiopulmonary bypass [15,20,21,25,26,28]. In their protocol, Li et al. [15] provided total priming fluid reduction to <1500 mL, retrograde oxygenated blood cardioplegia perfusion, modified ultrafiltration and albumin infusion to maintain a stable plasma colloid osmotic pressure. Gebauer et al. [25] primed the circuit with crystalloid solution, mannitol 20% (100 mL) and 100 mL of albumin 20%. Bypass flow was targeted to >3.2 l m2.min-1 and core temperature was lowered to 32–33 °C. For minimally invasive valve surgery, they routinely used crystalloid custodiol cardioplegia (>20–30 mL/kg) and, while on CPB, they routinely applied haemofiltration for the removal of the priming volume and cardioplegia targeting a zero to negative fluid balance. Similarly, other experiences reported a restrictive fluid substitution [21] and a reduction in total fluid priming to 900–1100 mL [26]. Zaouter et al. [20] and Berretta et al. [28] favoured the use of minimally invasive extracorporeal circulation (MiECC).

### 3.5. Postoperative Phase

#### 3.5.1. Pain Control

Three studies reported methods of postoperative pain evaluation including a Likert scale [22], the use of a numeric rating scale [23,27] and through the Behaviour Pain Scale in intubated patients [23].

Table 3 provides details about the pain management protocols as reported in the five studies that also evaluated postoperative opioid use [17,18,19,20,24]. After surgery, paracetamol was generally used as the first-choice therapy, while some experiences reported the use of ketamine [26], oxycodone [20], NSAIDs [22,25,26] or tramadol [28], with the systematic [22] or as-needed (VAS > 3) [26] addition of low doses of opioids.

Local [21] and locoregional anaesthesia [17,25,26,28] was continued until the first postoperative day in patients who underwent minithoracotomy surgery. Different protocols were proposed as concomitant therapies or in the case of breakthrough pain: the administration of fentanyl and acetaminophen [17] and the use of ketamine (0.05–0.15 mg/kg/h during the first 24 h) + paracetamol (1 g/6 h) + nefopam (20 mg IV/4 h during 2 days) + MgSO_4_ (3 g IV/24 h during 2 days) + ketoprofen (50 mg/6 h during 4 days if GFR > 60 mL/min/1.73 m^2^) + opioid [26], tramadol 4–8 μg/kg/min or morphine 10–20 mg/24 h [28].

#### 3.5.2. Early Physiotherapy and Mobilisation

Early physiotherapy and mobilisation after surgery were covered in twelve experiences [15,17,18,19,20,21,22,24,25,26,27,28]. Some protocols reported that physiotherapy and mobilisation should begin as soon as possible without further details regarding timing and clinical criteria [15,22,24].

Fleming et al. [18] described the first mobilisation as “sitting regularly in a chair from the first postoperative morning onwards”.

An early mobilisation starting from 2 to 6 h since the extubation was advocated by Hendy et al., “early mobilization starting with sitting at the edge of the bed for 4 h after tracheal extubation” [17]; Williams et al. [19], “when hemodynamically stable and extubated, the patient is assisted out of bed to a chair and activity advanced as tolerated to ambulation 4 times daily”; Zaouter et al. [20], “mobilization on chair on the same day after surgery”.

In the experience of Petersen et al. [21], all patients received their first postoperative physiotherapy treatment in the recovery room 2–3 h after the operation, which included breathing exercises and active mobilisation in the sitting and upright position. The patients were usually transferred to the ICU for overnight monitoring and received their second physiotherapy session in the evening, guided by ICU nursing staff.

Gebauer et al. [25,30] reported that all their patients received the first postoperative physiotherapy treatment in the PACU, two to three hours after surgery with passive mobilisation in bed, respiratory exercises and active mobilisation to the upright position. The patient was then encouraged to sit down at the edge of the bed with the consensus of the responsible anaesthetist and after a careful evaluation of the motoric and sensory functions of the upper and lower extremities as well as the level of postoperative pain. After performing intensive respiratory therapy in the sitting position, the mobilisation was continued to the upright position at the bedside and a first step.

Giger et al. [26] proposed early postoperative active and passive cardio-muscular and respiratory rehabilitation, including respiratory exercises after extubation (passive with spirometer and active with the physiotherapist), assuming a sitting position after extubation and active ambulation on the first postoperative day. Finally, Berretta et al. [28] provided a detailed pathway of early mobilisation and physiotherapy including respiratory therapy (3–6 h after surgery), early mobilisation (6–12 h after surgery) with bed exercises, bed and chair sitting, standing position and ambulation with the possibility of immediate patient–family contact.

#### 3.5.3. Lines and Chest Drains Removal

In order to facilitate mobilisation and to reduce the risk and the burden of perioperative infection, several experiences proposed an early removal of chest drains [15,20,21,22,24,25,26,28], urinary catheters [20,21,22,25,26] and venous and arterial lines [20,21,22,26]. Usually, drainage tubes were removed 12 h after surgery or on postoperative day 1 [15,21,25,28] or postoperative day 2 [22]. This was similar for arterial and venous lines and urinary catheters [21,22,25,26]. However, clear guidance on whether to remove or retain drains, lines and catheters has usually not been reported, with the exception of Zaouter et al. [20], who provided a detailed protocol with the removal of chest tubes when collecting <100 mL of blood in 8 h, the removal of urinary catheter if urinary output was >0.5 mL/h for 6 consecutive hours with no diuretic prescribed and the removal of the central venous line at discharge from the ICU.

#### 3.5.4. Oral Intake and Nausea/Vomiting Prevention

Four papers included the early resumption of oral intake starting within/from 6 h since extubation in their ERAS protocol [15,17,26,28]. A complete diet was resumed from postoperative day 1 or day 2 [15,22,28].

The prevention of nausea and vomiting represented a key action in the postoperative period in several experiences [15,16,17,18,19,22,25,26]. Different protocols were provided with the use of stat doses or the continuous infusion of i.v. ondansetron [15,16,18,19,25] and the adjunct of promethazine when needed [16,19], or with the prescription of droperidol [25,26].

### 3.6. Outcomes

Ten papers reported data on early mortality (in-hospital or 30-day mortality) [15,16,17,18,21,22,24,26,27,28] and showed no difference between patients who underwent surgery following an ERAS protocol and patients operated on following a conventional approach. Similarly, there was no difference in perioperative cerebral stroke [15,16,17,18,26,28] that generally occurred in less than 1% of the cases. Postoperative atrial fibrillation was registered in 4% to 39% of patients in the ERAS cohorts and in 11% to 34% following conventional treatment [15,16,17,18,20,22,24,25,26,28]. The study of Li et al. [15] reported a significant difference in the occurrence of postoperative AF: 3.8% in the ERAS population vs. 11.4% in the control group (*p* = 0.04).

Five studies [15,17,22,27,28] found a significant reduction in mechanical ventilation time in patients undergoing an ERAS pathway, with a median time ranging from 0 (on table extubation) to 23.5 h versus 6 to 272 h in the conventional treatment cohorts. Giger et al. [26] reported no difference in terms of mean mechanical ventilation time (ERAS 6.9 h vs. conventional 6.5; *p* = 0.86) but they reported a higher rate of extubation within the first 6 postoperative hours in patients in the ERAS group (87% vs. 65%; *p* < 0.001).

Eleven full texts provided details about length of ICU stay. Among them, nine studies [15,19,20,21,22,25,26,27,28] reported a significantly shorter stay in the ICU for patients operated on following an ERAS protocol with a mean stay of 18 to 30 h vs. 22 to 48 h for conventional surgery. On the contrary, Loria et al. [24] (3.1 days vs. 3.3 days, respectively) and Blitzer et al. [16] (6.9 days vs. 6.9 days, respectively) found no difference.

Eight studies [17,19,20,21,22,25,27,28] found a shorter hospitalisation time in patients in ERAS cohorts when compared with patients in control groups. The remaining six papers showed no difference [15,16,18,23,24,26].

Two full texts included details of the medical costs associated with ERAS and conventional approaches. Li et al. [15] found a lower cost for patients in the ERAS group, CNY 69,202 (52,089–123,823) vs. CNY 77,058 (51,390–144,290) in the control group (11% of difference, *p* = 0.002), and Petersen et al. [21] reported medical costs of EUR 11,200.0 ± 3030 per patient in the ERAS group and EUR 13,110 ± 4528 per patient in the control group, with a financial advantage derived from the implementation of the ERAS program of EUR 1910 per patient (*p* = 0.006).

Table 1 summarises the data regarding postoperative outcomes. Table 3 provides details about endpoint(s) and key findings associated with the ERAS approach. Table 4 reports details of postoperative opioid use.

## 4. Discussion

### 4.1. The Advent of ERAS in Cardiac Surgery

The ERAS approach aims to improve patient outcomes by optimising several strategies throughout the perioperative journey. This concept was introduced in 1997 by Henrik Kehlet, who suggested that incorporating multiple changes in the management of the surgical patients could significantly improve the surgical results. In 2001, the ERAS study group was formed with the aim of producing and interpreting the best available evidence to support the idea of fast-track surgery. The first consensus guidelines for colorectal surgery were published in 2005, and since 2010, the ERAS Society [31] has been dedicated to creating guidelines and promoting the broad implementation of ERAS pathways [32]. Although protocols to improve recovery after surgery are well established in general, breast and thoracic surgery, it was not until 2019 that the first consensus guidelines on ERAS in cardiac surgery became available [13]. In the subsequent years, a number of observational and RCT studies have reported satisfactory early results, reduced costs and increased satisfaction in cardiac surgery patients operated on following institutional ERAS protocols [15,19,22,30].

Previous reviews and meta-analyses included experiences mainly in CABG and OPCABG patients [33], reporting on both ERAS and fast-track pathways [34] or “ERAS-like” programmes that covered only some of the key actions of the perioperative management [35]. The purpose of our study was to provide an updated look at the current status of ERAS in heart valves surgery and to summarise the available evidence and results that could potentially support its wider implementation in daily clinical practice.

We have searched for all the experiences embedding an ERAS protocol and including surgical heart valve operations published within the last 10 years, from January 2015 to January 2024. Most of the retrieved abstracts related to non-cardiac surgery and we were able to select 14 articles that included patients operated on for heart valve disease. Ten of these papers were published during or after 2020; however, in six cases [15,18,19,20,27,28], an enhanced recovery pathway was already established before the publication of the 2019 ERAS guidelines in cardiac surgery as the first effort to systemise protocols and outcomes. Not surprisingly, our study showed high heterogeneity in the implementation of different key actions throughout the surgical journey. Twelve out of fourteen protocols involved multiple interventions from the preoperative to postoperative phase, while two studies reported actions that were limited to the management of intraoperative and postoperative analgesia [23] and postoperative care [27].

### 4.2. Communication and Prehabilitation

Ten studies [15,17,18,19,20,21,22,24,25,26] included a structured program of patients’ assessment and education through an in-person meeting or printed material. The goal of this intervention was to provide in-depth information regarding the pathology, the surgical care and the expectations of recovery. Making patients and their relatives familiar with the procedures and the hospital environments with a booklet describing every step of the protocol or a video describing the patient’s arrival in the operating room can decrease stress and reduce anxiety [20]. However, the inclusion of psychological assessment and support still appears limited, as it was only reported in two experiences from Li et al. [15] and Petersen et al. [22]. Near-future directions may see the progressive use of telemedicine in improving the preventative and pre-hospitalisation care of surgical candidates with electronic platforms that provide tailored information and guidance and encourage engagement in appropriate physical exercise.

Despite previous studies in non-cardiac surgery, although in the presence of a heterogeneous offering of treatment/exercises and delivery methods, having demonstrated some benefits of a pre-admission period of rehabilitation—prehabilitation—in terms of reducing the length of stay in the ICU [36] and postoperative complications [37,38], there is no univocal evidence regarding its efficacy. Prior experiences with cardiac surgery patients showed that a prehabilitation program was also feasible for patients scheduled for CABG surgery [39] and was associated with reduced ICU and hospital lengths of stays, faster postoperative recovery and improved postoperative quality of life [40,41]. Recent ERAS guidelines stated that a prehabilitation period “*enables patients to withstand the stress of surgery by augmenting functional capacity. Preoperative exercise decreases sympathetic overreactivity, improves insulin sensitivity, and increases the ratio of lean body mass to body fat. It also improves physical and psychological readiness for surgery, reduces postoperative complications and the length of stay, and improves the transition from the hospital to the community. A cardiac prehabilitation program should include education, nutritional optimization, exercise training, social support, and anxiety reduction, although current existing evidence is limited*” [13]. Despite these recommendations, only six [17,19,20,21,25,26] of the fourteen studies offered a prehabilitation program aimed at improving the patient’s physical fitness and providing training to facilitate postoperative physiotherapy.

### 4.3. Cardiopulmonary Bypass Management

Extracorporeal circulation remains a major determinant of patient outcomes. No recommendations on the management of CPB have been incorporated into the ERAS guidelines for cardiac surgery [13], while a more recent multidisciplinary consensus [29] suggested that goal-directed perfusion may play a role in preventing organ injury associated with cardiopulmonary bypass and that haemodilution and blood transfusion can be prevented by retrograde autologous priming and priming volume reduction. Our study demonstrated that only a minority of experiences with ERAS in valve surgery involved key actions addressing the optimisation of cardiopulmonary bypass conduit. Noteworthily, only two studies [20,28] reported the use of minimally invasive extracorporeal circulation (MiECC). MiECC combines several strategies including the prevention of haemodilution, limitation of cardiotomy aspiration, active normothermia and technologies to increase hemocompatibility and anticoagulation monitoring through a combined surgical approach, anaesthesiology and perfusion management. A recent position paper by the MiECTiS Society [42] stated that MiECC can reduce haemodilution, postoperative bleeding and the need for red-blood-cell transfusion. Alongside improved myocardial and organ protection, the use of MiECC systems has been associated with a reduced rate of postoperative atrial fibrillation, preserved neurocognitive function and attenuated inflammatory response [42] and can promote the adoption of ultra-fast-track pathways [43]. Despite progressive refinements to increase its safety and adoption, minimally invasive extracorporeal circulation remains a demanding procedure requiring multidisciplinary management by surgeons, anaesthetists and perfusionists.

### 4.4. Analgesia, Anaesthesia and Early Extubation

Pain control after surgery is essential for patient comfort, early extubation and mobilisation and contributes to reducing complications and facilitating postoperative recovery [44,45]. The administration of parenteral opioids has been the cornerstone in pain management after cardiac surgery; however, their use is associated with multiple side effects such as oversedation, respiratory depression and postoperative ileus. Multimodal opioid-reducing/sparing analgesia with the use of more than one pharmacological class of analgesic drugs, targeting different receptors along the pain pathway, aims at improved pain control while reducing adverse effects associated with each class of medication, especially those of opioids [17,18,19,20,23,24]. In most ERAS protocols, paracetamol (acetaminophen) represents the first-line medication for pain control after surgery because it does not interfere with the bowel function and provides superior analgesia when associated with low-dose opioids [13]. According to the ERAS guidelines, in cardiac surgery “*there is sufficient evidence to recommend that cardiac surgery programs use acetaminophen, tramadol, dexmedetomidine, and pregabalin (or gabapentin) based on formulary availability*”.

Despite the fact that adjuncts of loco-regional analgesia can be a valid tool for perioperative pain control after cardiac surgery [46], the cardiac ERAS guidelines do not provide any specific recommendation for their implementation [13]. Data on regional analgesia are extremely limited in the context of cardiac surgery, as they come from small sample sizes with high heterogeneity in techniques and the measurement of outcomes. Nevertheless, the available evidence indicates that loco-regional analgesia could facilitate enhanced recovery pathways [15,20,26,28,29,45,47]. The recent joint consensus from the ERAS Cardiac Society, the ERAS International Society and the Society of Thoracic Surgeons (STS) recommended chest wall regional analgesia as “*an effective component of a multimodal approach to perioperative pain management*” [29] and suggested that further research is required to establish delivery methods and the efficacy of locoregional pain control in cardiac surgery.

The adoption of fast-track cardiac anaesthesia with short-acting narcotic and sedative agents is advocated to reduce the time of mechanical ventilation [15,17,19,22,25,28,48]. Although early extubation represents one of the main goals in most experiences that include an ERAS program [17,19,20,21,22,25,26,27,28], the results of our review showed that there was wide heterogeneity in the duration of postoperative mechanical ventilation time, with some studies reporting a mean postoperative intubation time between 0 and 10 h [15,17,18,22,24,26,28], whereas others included patients with an average intubation time between 1 day and 3 days [16,27].

Several studies have already underscored the benefits associated with early/fast-track extubation, especially in OPCABG and CABG operations, mini heart valve surgery and transapical TAVI [10,11,49,50,51]. Two recent observational studies on minimally invasive mitral valve surgery showed that early extubation (within 6 to 10 h after the end of the surgical procedure) was associated with reduced stays in intensive care units and reduced overall hospitalisation [45,51]. A further step is represented by on-table extubation in the operating room at the end of the surgical procedure. Despite earlier concerns about safety issues [52], more recent evidence showed that on-table extubation can be safely achieved in cardiac surgery patients with no increased risk of reintubation for respiratory failure. Moreover, on-table extubation was shown to be associated with a further shortening of ICU and hospital stays [25,45,53,54,55]. Based on this growing evidence, the ERAS guidelines recommend strategies to ensure extubation within 6 h of surgery [13,29].

### 4.5. Early Mobilisation

One of the advantages associated with early extubation is the possibility to start respiratory therapy and to mobilise the patient as soon as possible. In non-cardiac surgery specialities, early ambulation and physiotherapy have been associated with reduced intensive care unit stays and better mobility at the time of discharge [56,57,58]. In cardiac surgery, early mobilisation can improve pulmonary function and reduce ICU lengths of stays, while promoting a better exercise capacity and a quicker functional recovery. Improved functional capacity is associated with a higher rate of home discharge without the need for any further period of rehabilitation [40,59,60].

The interpretation of the results coming from the literature remains difficult. As already highlighted in this review, there are no consistent details on the timing of early mobilisation; furthermore, clear eligibility criteria for this action are missing. According to the proposed protocols and keeping in mind the limitation of having selected mostly young/middle-aged low-risk patients, a safe approach under the care of physiotherapists and after a clinical evaluation by anaesthetists or intensivists would suggest starting “early physiotherapy” after 2–6 h from tracheal extubation [17,21,25,26,28]. Controlled breathing exercises (active or passive), bed activities with upper and lower limb exercises, followed by bed-sitting at three to six hours after surgery and mobilisation with upright position after 12 h or on postoperative day 1 seem to be perfectly safe.

Many factors can impact achieving early mobilisation, such as pain control, the need for intravenous fluid or medication support and the presence and maintenance of chest tubes or lines. Only one study [20] provided clear guidance regarding the decision of whether to remove or maintain chest tubes. The remaining experiences stated that usually, the drainage tubes and other lines were removed between 12 h after surgery and postoperative day 2 [15,21,22,25,26,28]. Another noteworthy finding was the limited availability of details regarding protocols for postoperative pain control and methods of pain assessment, with very little data on the means and timing of postoperative pain assessment.

All studies that included a structured protocol of early physiotherapy and mobilisation were characterised by shorter times of mechanical ventilation [17,28] and by reduced lengths of ICU [20,21,25,26,28] and hospital stays [17,20,21,25,28]. These actions are interdependent objectives and further highlight the importance of the ERAS concept of introducing multiple key actions which can act synergically to improve surgical outcomes. However, the positive impact of these interventions on mortality and major postoperative morbidity has not yet been clearly demonstrated by different authors. As already highlighted before, the lack of clear evidence may be due to the large inclusion of young, low-risk patients with an overall low incidence of adverse events.

### 4.6. The Role of Minimally Invasive Cardiac Surgery

Noncardiac surgery ERAS guidelines support, with a strong grade of recommendation, minimally invasive access in gynaecologic surgery as “*an important tenet of ERAS*” and an option to be “*preferred for appropriate patients when feasible*” [57]. Similarly, for general surgery, based on high-level evidence and a strong grade of recommendation, ERAS guidelines state that “*a minimally invasive approach to colon and rectal cancer has clear advantages for improved and more rapid recovery, reduced general complications, reduced wound-related complications including incisional hernia and fewer adhesions. It is also an enabler for successful administration of many of the major components of ERAS such as opiate sparing analgesia and optimised fluid therapy*” [58]. On the contrary, cardiac ERAS guidelines [13] do not provide any specific recommendation or suggestion regarding the adoption of minimally invasive surgery to improve patients’ outcomes and recovery, although a smaller incision, the avoidance of sternotomy and reduced surgical trauma could intuitively facilitate pain control, early mobilisation and lower wound complications. Heart valve surgery through reduced surgical access (mini-thoracotomy, mini-sternotomy) is nowadays well established in highly specialised heart valve centres and has proven to be as safe and effective as conventional treatment [61,62]. However, recent national and multi-institutional data showed that minimally invasive cardiac surgery techniques are not widely adopted and that mini-thoracotomy mitral surgery is performed only in 23% to 54% of the patients scheduled for mitral valve procedures [1,63,64,65]. Not surprisingly, only four studies in this review included patients operated on with minimal access approaches [20,21,25,28]. Reduced surgical access may be perceived as being difficult for adequate exposure and myocardial protection and can translate into longer cardiopulmonary bypass and cross-clamp times. Tissue trauma is important and undoubtedly affects the postoperative recovery; however, lengthy and complex procedures based on the only perspective of providing cardiac operations through small cuts can hardly be seen as minimally invasive surgery since biological trauma and operative times remain the main determinant of perioperative complications.

Recent experiences have highlighted that minimally invasive heart valve surgery, when integrated in an enhanced recovery after surgery pathway, while providing safe operations and durable results, can accomplish patients’ desire for easier mobilisation, faster recovery and return to normal activity, as it has been associated with reduced mechanical ventilation time and shorter ICU stays and hospitalisation times [45,51,66,67]. Nowadays, having achieved excellent results in terms of low mortality and low postoperative complications, minimally invasive cardiac surgery is called upon to address these new therapeutic targets that are of the utmost importance both for young and active patients who may experience the perioperative journey as the worst health issue that affects their lives, and for elderly and comorbid patients who could still benefit more from attenuated physical, physiological and psychological trauma.

## 5. Conclusions

In the context of heart valve surgery, there is limited evidence regarding the implementation and benefits of enhanced recovery after surgery pathways. Our study summarised relevant findings from initial ERAS experiences in cardiac surgery, which demonstrated its feasibility and potential for improving surgical outcomes in the treatment of patients undergoing heart valve operations. Multidisciplinary teamwork represents the core of the ERAS approach and allows the promotion of key actions for preoperative (education, prehabilitation, diet improvement), intraoperative (multimodal analgesia, fast-track anaesthesia, CPB conduit) and postoperative (pain control, early extubation, early mobilisation and feeding) management.

Despite the lack of impact of the ERAS programmes on early mortality and postoperative morbidity, these protocols have proven to be safe and were associated with shorter mechanical ventilation time, reduced ICU and hospital stays and reduced postoperative opioid use.

## 6. Future Directions

The present day is still to be considered the dawn of the ERAS approach in cardiac surgery. Next steps should include a broader implementation of ERAS protocols leading to the increased recruitment of patients and hence the availability of larger data sets to demonstrate feasibility and associated benefits. Further studies should focus on the impact of different surgical accesses and on the management of cardiopulmonary bypass. Additional patient-reported outcomes, beyond the usual hard endpoints, should be targeted in future studies to evaluate the impact of ERAS in cardiac surgery. It would be of utmost importance to address the evaluation of postoperative pain and patient satisfaction, assessing quality of life, recovery and psychological status. The inclusion of frail and higher-risk patients could further reveal the advantages to the prognosis and morbidity of a shared multidisciplinary ERAS approach in this less-healthy group of patients.

## Figures and Tables

**Table 3:** Study endpoints and principal findings.

Author(s), Year	Primary Endpoint(s)	Secondary Endpoint(s)	Key Findings Associated with ERAS Management (*p* < 0.05)
Li et al., 2018 RCT [15]	Time to readiness for hospital discharge	Length of ICU stay Time to first bowel movement Hospitalisation costs The duration of postoperative mechanical ventilation Use of vasoactive drug support Time to first full diet Time to drainage tube removal Visual analogue scale pain score Results of major laboratory tests Adverse events during hospitalisation Long-term (6 months) complications and patient satisfaction	Reduced postop AF Reduced MV time Reduced ICU stay Reduce time of vasoactive drug support Readiness to discharge Reduced time to first bowel Reduced time to drain removal Reduced medical cost
Blitzer et al., 2022 OS [16]	Postoperative pain control		
Hendy et al., 2022 OS [17]	Hospital stay		Reduced MV time Reduced time to ambulation Reduced hospital stay Reduced opioid consumption
Fleming et al., 2016 OS [18]	Length of stay	Perioperative outcomes	Reduced complications Reduced time of postop oral intake Reduced pain Reduced opioid consumption
Williams et al., 2019 OS [19]	Opioid use Gastrointestinal complications Length of stay Patient and staff satisfaction		Reduced ICU stay Reduced hospital stay Reduced GI complications Reduced opioid consumption
Zaouter et al., 2019 OS [20]	Length of stay Postoperative outcomes		Reduced ICU stay Reduced hospital stay Reduced pain
Petersen et al., 2021 OS [21]	Medical costs		Reduced ICU stay Reduced hospital stay Reduced medical cost
Yazdchi et al., 2021 OS [22]	Total ventilation times Length of intensive care unit stay Postoperative hospital length of stay	Postoperative complications Hospital mortality 30-day readmission rates	Reduced MV time Reduced ICU stay Reduced hospital stay
Bills et al., 2022 OS [23]	Opioid consumption	Pain scores Length of stay Opioid-related complications	Reduced opioid-related complications
Loria, 2022 OS [24]	Opioid consumption		Earlier chest tube removal Reduced opioid consumption
Gebauer et al., 2023 OS [25]	ICU length of stay Postoperative hospital length of stay Postoperative complications		Reduced ICU stay Reduced hospital stay
Giger et al., 2023 OS [26]	Hospital mortality Hospital morbidity Length of stay Blood management		Reduced ICU stay Reduced MV time Reduced postop delirium Reduced AKI Reduced chest infection Reduced transfusion
Obafemi et al., 2023 OS [27]	Hospital mortality Hospital morbidity ICU length of stay Hospital length of stay Time until extubation Time until urinary catheter removal		Reduced ICU stay Reduced MV time Reduced hospital stay Reduced time to first ambulation
Berretta et al., 2023 OS [28]	Hospital mortality Postoperative cerebral event	Minor complications Length of stay Survival	Reduced ICU stay Reduced MV time Reduced hospital stay Reduced respiratory insufficiency Reduced ICU readmission

**Table 4:** Postoperative opioid use.

Author(s), Year	Patients	Analgesia Management	Opioid Use	*p*
Hendy et al., 2022 OS [17]	100 ERAS	Pre-emptive analgesia: acetaminophen (650 mg) and gabapentin (300 mg) 30 min before anaesthesia Ultrasound-guided serratus anterior plane block is performed within one hour from arrival to ICU. Fentanyl (25 mcg) is delivered directly through IV injections every 5 min, as needed (with a maximum of 300 mcg over 6 h) and/or acetaminophen (1000 mg) every 6 h, not to exceed 4 g/day if the nerve block was not sufficient.	11.58 ± 4.43 morphine milligram equivalent (ICU consumption)	<0.001
	103 control	From the time of arrival to the ICU until extubation, fentanyl is infused at 25–100 mcg/h. After extubation, fentanyl (25 mcg) is delivered directly through IV injections every 5 min, as needed (with a maximum of 300 mcg over 6 h). Hydromorphone (1–2 mg) is delivered subcutaneously every 3 h as needed, and 2–4 mg orally every 3 h as needed. Non-opioid adjuvants. Acetaminophen (1000 mg) every 6 h, not to exceed 4 g/day. Ketorolac (15 mg) is given intravenously every 8 h (not given if bleeding or if the patient has abnormal kidney function)	50.58 ± 11.93 morphine milligram equivalent (ICU consumption)	
Fleming et al., 2016 OS [18]	52 ERAS	Gabapentin, 600 mg PO preoperatively Opioid (morphine) infusion discontinued after extubation Analgesia after extubation: regular paracetamol and codeine with additional oral solution of morphine sulphate, if needed	Opioid infusion duration (days): 0 (0–0)	<0.01
	53 control		Opioid infusion duration (days): 3 (2–3)	
Williams et al., 2019 OS [19]	443 ERAS	Preoperative: gabapentin (300 mg) and acetaminophen (1000 mg) given orally in preoperative holding area Intraoperative: fentanyl IV given as needed for pain but typically <1 mg for entire case Hydromorphone (0.5–1 mg) given near completion of surgery If time since preoperative acetaminophen dose significantly exceeds 6 h, 1 dose of acetaminophen (1000 mg) IV considered Postoperative acetaminophen (1000 mg) every 6 h Gabapentin (300 mg) twice daily, weaned after POD 5 Oxycodone (5–10 mg) every 4 h as needed (liquid given through orogastric tube while intubated, orally once extubated and tolerating clears) Fentanyl IV for breakthrough pain resistant to oral medication management	Mean 21 milligrams of intravenous morphine equivalent	<0.01
	489 control		Mean 29 milligrams of intravenous morphine equivalent	
Zaouter et al., 2019 OS [20]	23 ERAS	Intraoperative: pre-emptive multimodal analgesic strategy was implemented at induction and consisted of boluses of 0.5 mg/kg of ketamine After sternum closure, wound infiltration with a total of 20 mL of 0.75% ropivacaine was applied along with administration of a multimodal analgesia encompassing 1 g of acetaminophen, 100 mg of ketoprofen, 0.3 mg/kg of nefopam and 0.1 mg/kg of morphine Postoperative: patient-controlled analgesia morphine and nefopam (65 mg/kg/h) and pregabalin (150 mg) once a day for the first 5 PODs. When patients were discharged from the ICU, 100 mg of ketoprofen was prescribed twice a day with breakthrough and 100 mg of tramadol every 4 to 6 h, as required	2 (0–12) total mean milligrams of intravenous morphine equivalent	*p* = 0.09
	23 control	Intraoperative: target-controlled infusion with either sufentanil or remifentanil; during sternum closure using 0.2 mg/kg of morphine, 1 g of acetaminophen, and 0.3 mg/kg of nefopam when not contraindicated Postoperative: patient-controlled analgesia morphine (containing 0.05 mg of droperidol for each milligram of morphine) and 65 mg/kg/h of nefopam for the first 48 postoperative hours or until discharge from ICU. When patients were discharged from the ICU, 100 mg of ketoprofen was prescribed twice a day with breakthrough and 100 mg of tramadol every 4 to 6 h, as required	7 (3–12) total mean milligrams of intravenous morphine equivalent	
Bills et al., 2022 OS [23]	133 ERAS	No preoperative therapy Postoperative: acetaminophen (1000 mg) every 8 h; gabapentin (100–300 mg) every 8 h; methocarbamol (250–500 mg) every 6 h for 5 days with the option of extending therapy or making dose adjustments based on renal function, as well as tolerability and response. Lidocaine patches are also commonly added in these patients for relief of pain and ketorolac is occasionally used for breakthrough pain in patients with normal renal function	75.8 (40.6–128.7) cumulative oral mean milligrams of intravenous morphine equivalent (72 h)	*p* = 0.09
	185 control		105.4 (37.9–165.0) cumulative oral mean milligrams of intravenous morphine equivalent (72 h)	
Loria, 2022 OS [24]	216 ERAS	Preoperative Acetaminophen (1 g) 2 h before surgery Gabapentin (300 mg) 2 h before surgery Intraoperative Recommend reduced opioid use to <500 mg fentanyl Local anaesthetic with liposomal bupivacaine 10 mL chest tube sites 15 mL incision Postoperative Dexmedetomidine (initiated in OR, continued until extubation) Acetaminophen (650 mg) scheduled every 6 h Lidocaine 5% transdermal patch, applied to bilateral back or chest Gabapentin, 100 mg TID, 100 mg BID if renal impairment Tramadol, 50–100 mg PO every 6 h PRN for mild pain (every 12 h for CrCl < 30, max 200 mg/d) Oxycodone, 5–10 mg PO every 4 h PRN for moderate pain IV opioids (eg, hydromorphone, fentanyl), PRN severe/breakthrough pain	261 milligrams of intravenous morphine equivalent	*p* < 0.001
	250 control		459 milligrams of intravenous morphine equivalent	

## Data Availability

The original contributions presented in the study are included in the article/Supplementary Materials; further inquiries can be directed to the corresponding author.

## Supplementary Materials

The following supporting information can be downloaded at: https://www.mdpi.com/article/10.3390/jcm13102903/s1, Figure S1: Flow chart of literature search according to PRISMA guidelines; Table S1: PRISMA 2020 checklist [68].
